# A Rare Case of Disseminated Histoplasmosis in an Adult With Alcoholic Liver Cirrhosis

**DOI:** 10.7759/cureus.39744

**Published:** 2023-05-30

**Authors:** Marko Kozyk, Kateryna Strubchevska, John Szela

**Affiliations:** 1 Internal Medicine, Beaumont Hospital, Royal Oak, USA; 2 Infectious Disease, Beaumont Hospital, Royal Oak, USA

**Keywords:** histoplasma capsulatum, fungus, blastomycosis, alcoholic liver cirrhosis, disseminated histoplasmosis

## Abstract

Histoplasmosis, also known as "Darling’s disease," is caused by the dimorphic fungus *Histoplasma capsulatum,* which is distributed all over the world but is more common in Northern America. In this paper, we present the case of an adult patient with decompensated liver cirrhosis who had positive antigen test results for *H. capsulatum* and *Blastomyces dermatitidis*. Disseminated histoplasmosis was confirmed by means of additional antibody testing in a patient with septic shock complicated by multiorgan failure and duodenal perforation. A high index of suspicion is required for the detection of disseminated histoplasmosis.

## Introduction

*Histoplasma capsulatum* and *Blastomyces dermatitidis* are dimorphic fungi that cause subclinical, self-limited, or disseminated infections. Clinical manifestations and geographic distribution are very similar between these two pathogens, which makes it challenging to differentiate one infection from another [1]. In addition, cross-reactivity between Histoplasma and Blastomyces antigens has also been established [2]. The association of infection with specific occupational risk factors and additional antibody testing allow physicians to make the correct diagnosis. Even though visualization of the yeast in a culture is a gold standard method of diagnostics, helping to distinguish two fungi, it may take weeks [1].

## Case presentation

A 58-year-old man with a past medical history of alcoholic liver cirrhosis, hypertension, and gastroesophageal reflux disease presented to the emergency department with jaundice and shortness of breath for five days. His vital signs on admission were within the normal range. The physical exam revealed jaundice, bilateral hand tremor, hepatomegaly, and bilateral lower extremity pitting edema. His pertinent laboratory results are shown in Table 1.

**Table 1:** Laboratory results.

White blood cell count	12.3 × 10^3 ^(reference range, 4.5–11)/µL
Hemoglobin	7.8 (reference range, 13.8–18) g/dL
Platelet count	79 (reference range, 150–400) × 10^3^/µL
Alkaline phosphatase	264 (reference range, 44–147) U/L
Aspartate transaminase	90 (reference range, 8–33) U/L
Total bilirubin	21.5 (reference range, 0.1–1.2) mg/dL
Direct bilirubin	11 (reference range, less than 0.3) mg/dL
Creatinine	1.5 (reference range, 0.7–1.1) mg/dL
BUN	30 (reference range, 6–24) mmol/L
ESR	30 (reference range, 0–15) mm/hr

The chest radiographs demonstrated diffuse interstitial infiltrates bilaterally. He was empirically treated for pneumonia with ceftriaxone and azithromycin. Of note, the patient worked as a traveling construction laborer at outdoor facilities in the Midwest.

**Figure 1:**
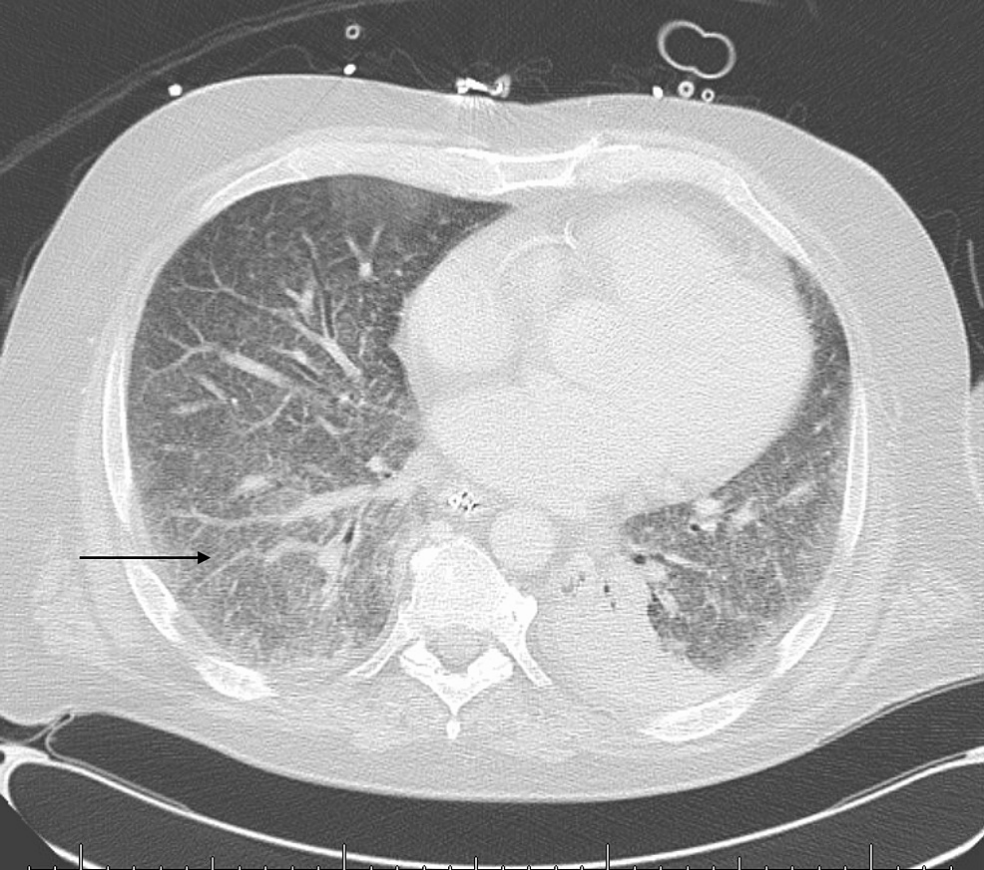
CT scan of the chest.

His hospital course was complicated by septic shock, worsening mentation, and increasing oxygen requirements, leading to a transfer to the intensive care unit (ICU) and intubation. The treatment team was concerned about central nervous system involvement, and a lumbar puncture was performed. Cerebrospinal fluid (CSF) findings included an elevated white cell count, elevated protein, and low glucose. The patient underwent an extensive infectious disease workup for bacterial and viral pathogens, which was negative. Bronchoalveolar lavage, urine, blood, and CSF tests were positive for Histoplasma capsulatum and Blastomyces dermatitidis antigens (MiraVista Diagnostics, Indianapolis, IN). Histoplasma antibodies (M precipitation bands) were positive, while Blastomyces antibodies were negative (ARUP Laboratories, Salt Lake City, UT). The patient was started on liposomal amphotericin B, meropenem, and daptomycin. A CT scan of the chest revealed extensive bilateral ground glass opacities in the lungs with focal consolidation in the left lower lobe and left upper lobe as well as hepatosplenomegaly (Figure 1).

A CT scan of the abdomen showed a complex peritoneal air-fluid collection in the right upper quadrant and free air, indicating a perforated viscus. An exploratory laparotomy was performed and revealed duodenal perforation as well as peritonitis. Peritoneal fluid cultures were positive for Enterococcus faecium and vancomycin-resistant Enterococci. The patient stayed in the ICU for three weeks and developed distributive shock with multiorgan failure. He had acute renal failure; therefore, continuous renal replacement therapy was started. Bilious return was noted from endotracheal tube suctioning with increasing ventilation requirements and desaturation. The patient had a very poor prognosis, and the family switched to comfort care measures. The time of expiration was 17 days after his hospitalization.

## Discussion

Histoplasmosis is caused by the thermally dimorphic environmental fungus H. capsulatum, which is distributed worldwide and associated with river valleys. In the United States of America, histoplasmosis is endemic in the Ohio and Mississippi River valleys [1].

Under natural conditions, the causative agent of histoplasmosis primarily lives in moist soil, bird droppings, and bat droppings. Infection in humans occurs after inhalation of spores. Occupations related to demolition, construction, and excavation in endemic regions are particularly associated with histoplasmosis.

Histoplasmosis is difficult to diagnose because of its variable clinical manifestations, ranging from asymptomatic to disseminated infection, and the absence of pathognomonic features. Patients with impaired cellular immunity, such as those with AIDS, hematologic oncology, or transplant recipients, are particularly at high risk for developing disseminated histoplasmosis [1]. However, our patient had alcoholic liver cirrhosis, which might also have some association with the disseminated disease. Also, he worked at outside construction sites, which demonstrates that clinicians should always consider histoplasmosis as a possible diagnosis in patients with certain occupational risk factors.

Obtaining positive culture results is usually a time-consuming process; therefore, antigen and antibody detection, as well as molecular testing, are performed first due to faster test results. Histoplasma antigen testing has a sensitivity of 55-67.5%, and Histoplasma antibody testing is 67.5-87.5% sensitive; however, a combination of Histoplasma capsulatum antigen and antibody testing increases the sensitivity to 96% [2]. H. capsulatum and B. dermatitidis antigens were positive in our patient’s cerebrospinal fluid, blood, bronchoalveolar lavage, and urine. It is unusual to get both H. capsulatum and B. dermatitidis infections at the same time. Cross-reactivity between these antigens is very common. For example, 63.2% of positive urinary histoplasmosis antigen assays cross-reacted with Blastomyces urinary antigen assays [3]. Based on current data, a comparison between Histoplasma and blastomycosis antibody titers should be done to determine which fungus is the real culprit. In our case, additional antibody testing favored the diagnosis of disseminated histoplasmosis [4]. Liposomal amphotericin B remains the antifungal drug of choice for the initial treatment of severe disseminated histoplasmosis [5].

## Conclusions

In this case report, we illustrated that it is common to have cross-reactivity between H. capsulatum and B. dermatitidis antigen test results. These pathogenic dimorphic fungi have similar manifestations and are very common in the Midwestern USA. Prompt identification as well as a high index of suspicion are necessary for the proper diagnosis and management of disseminated histoplasmosis. Clinicians need to be aware of the possibility of disseminated histoplasmosis even in immunocompetent patients, as this can be very helpful in making the correct diagnosis and providing appropriate treatment.
